# Using whole-genome sequencing (WGS) to plot colorectal cancer-related gut microbiota in a population with varied geography

**DOI:** 10.1186/s13099-022-00524-x

**Published:** 2022-12-28

**Authors:** Han Shuwen, Wu Yinhang, Zhao Xingming, Zhuang Jing, Liu Jinxin, Wu Wei, Ding Kefeng

**Affiliations:** 1 Department of Colorectal Surgery and Oncology, Key Laboratory of Cancer Prevention and Intervention, Ministry of Education, The Second Affiliated Hospital, Zhejiang University School of Medicine, 88 Jiefang Road, Building 6 Room 2018, Hangzhou, 310009 Zhejiang China; 2 Cancer Center Zhejiang University, Hangzhou, Zhejiang China; 3 Huzhou Central Hospital, Huzhou, Zhejiang China; 4 Institute of Science and Technology for Brain-Inspired Intelligence, Fudan University, Shanghai, China

**Keywords:** Colorectal cancer, Intestinal bacteria, Regional difference

## Abstract

**Background:**

Colorectal cancer (CRC) is a multifactorial disease with genetic and environmental factors. Regional differences in risk factors are an important reason for the different incidences of CRC in different regions.

**Objective:**

The goal was to clarify the intestinal microbial composition and structure of CRC patients in different regions and construct CRC risk prediction models based on regional differences.

**Methods:**

A metagenomic dataset of 601 samples from 6 countries in the GMrepo and NCBI databases was collected. All whole-genome sequencing (WGS) data were annotated for species by MetaPhlAn2. We obtained the relative abundance of species composition at the species level and genus level. The MicrobiotaProcess package was used to visualize species composition and PCA. LEfSe analysis was used to analyze the differences in the datasets in each region. Spearman correlation analysis was performed for CRC differential species. Finally, the CRC risk prediction model was constructed and verified in each regional dataset.

**Results:**

The composition of the intestinal bacterial community varied in different regions. Differential intestinal bacteria of CRC in different regions are inconsistent. There was a common diversity of bacteria in all six countries, such as *Peptostreptococcus stomatis* and *Fusobacterium nucleatum* at the species level. *Peptostreptococcus stomatis* (species level) and *Peptostreptococcus* (genus level) are important CRC-related bacteria that are related to other bacteria in different regions. Region has little influence on the accuracy of the CRC risk prediction model. *Peptostreptococcus stomatis* is an important variable in CRC risk prediction models in all regions.

**Conclusion:**

*Peptostreptococcus stomatis* is a common high-risk pathogen of CRC worldwide, and it is an important variable in CRC risk prediction models in all regions. However, regional differences in intestinal bacteria had no significant impact on the accuracy of the CRC risk prediction model.

**Supplementary Information:**

The online version contains supplementary material available at 10.1186/s13099-022-00524-x.

## Introduction

Cancer incidence and death rates are increasing worldwide. The GLOBOCAN 2020 database showed that lung cancer incidence is high in East Asia, Eastern Europe and southern Europe, nonmelanoma skin cancer incidence is highest in the United States, Australia and Canada, cervical cancer incidence is high in Central Africa, and breast cancer incidence is the highest in most other countries [1]. Cancer is a multifactorial disease, and current research is focused on determining the risk factors for cancer. Cancer risk factors can be classified as genetic and environmental [2]. Different distributions of cancer risk factors, especially environmental factors and geographical differences, are the main factors that cause the difference in the highest incidence of cancer in different countries.

Globally, CRC ranks third in incidence and second in mortality among all malignancies [3]. The incidence of CRC increases with the level of economic development [4]. According to statistics, the incidence of colon cancer varies approximately 9 times in different regions of the world. Northern Europe has the highest incidence (25.3% for men and 16.4% for women). The incidence of rectal cancer has a similar regional distribution, and the lowest incidence was found in East Asia (2.8% for men and 1.9% for women). Overall, the incidence of CRC is low in most parts of Africa and Central and South Asia (all less than 9.0%) [1]. In addition, the incidence and mortality rates of CRC vary by race, with black individuals having the highest rates (45.7%) and Asians having the lowest (30.0%) [5].

CRC is a cancer caused by a combination of genetic and environmental factors. For example, RAS [6] and BRCA [7] gene mutations and mismatch repair genes MLH1, MSH2, MSH6, and PMS2 mutations [8] are associated with CRC risk. Most CRCs are sporadic cancers caused by environmental factors, among which the main risk factors include obesity, physical inactivity, poor diet, alcohol consumption and smoking [9]. Regional and population differences in CRCs lead to regional differences in cancer-related risk factors, including socioeconomic factors, diet, intestinal microbial changes, immune microenvironment changes, and genetic mutations [10]. In addition, the coverage ratio of cancer screening and targeted intervention measures are important factors in influencing the regional difference in CRC incidence [11]. Genome-wide sequencing association studies identified risk-associated loci for CRC, but these loci did not differ significantly between regions [12, 13]. Existing studies are still unable to account for regional variations in CRC risk.

There is considerable evidence that microbial dysregulation in the human gut is an important risk factor for CRC. Decrease in propbiotic and increase in pathogenic bacteria was identified in CRC incidence. Probiotics (such as *Bifidobacterium*, *Lactobacillus* and *Bacteroidetes*) decreased, while pathogenic bacteria (such as *enterotoxigenic Bacteroidetes*, *Escherichia coli* and *Clostridium difficile*) increased [14]. The main causes of gut microbiota dysbiosis include diet, drugs, environmental pollutants and gut immune dysfunction and so on [14–16]. For example, Chen et al. reported that most patients with advanced colorectal adenoma had a low fiber diet. Meanwhile, the abundance of *Clostridium*, *Roseburia*, and *Eubacterium spp.* in the advanced colorectal adenoma group with a low fiber diet increased, while the abundance of *Enterococcus*, *Streptococcus spp.* and butyric-producing bacteria decreased [17]. Increased toxins secreted by the bacteria lead to intestinal mucosal damage and chronic inflammation, which ultimately induces CRC [18]. Intestinal microbes are directly affected by the genetic and environmental CRC risk factors mentioned above [19].

In environmental risk factors, for example, obesity may contribute to CRC through LPS-mediated systemic inflammation and a decrease in short-chain fatty acid (SCFA)-producing bacteria [20]. High-fat diets induce intestinal microecological disorders, leading to an increase in pathogenic bacteria (*Alistipes sp. Marseille-P5997*, *Alistipes sp. 5CPEGH6*) and a decrease in beneficial bacteria (*Parabacteroides distasonis*, *Parabacteroides sp. CT06*). Finally, it damages intestinal barrier dysfunction and promotes the occurrence of CRC [21]. High-fiber diets inhibit CRC by increasing bacterial metabolites that produce SCFA [22]. Long-term alcohol consumption or smoking can reduce the abundance of *Bifidobacteria*, *Bacteroides* and *Firmicute*s and increase the abundance of *Proteus* and *Actinomycetes* [23, 24]. Genetic mutations also play a role in differences in gut microbial composition and abundance. Mutation of the KRAS gene can change the abundance of *Roseburia*, *Parabacteroides*, *Metascardovi*a, *Staphylococcus*, and *Bacillale* and affect the composition of the intestinal bacterial community [25]. Mutation of APC is closely related to changes in intestinal microbiota (such as *Faecalibacterium prausnitzii* and *Fusobacterium mortiferum*) and serum metabolites [26]. Some intestinal microorganisms can also inhibit the inhibitory effect of P53 on the WNT pathway and promote the occurrence of CRC [27]. Importantly, gene mutations of CRC are also affected by region or ethnicity [28]. Yong et al. found that BRAF and KRAS mutation rates in CRC in Asian patients were lower than those in North American CRC patients [29]. Guda et al. found 15 novel mutated genes in African-American patients with CRC, which are rarely mutated in Caucasians with CRC [30]. Therefore, differences in CRC risk factors (both genetic and environmental) caused by regional or geographical factors can directly lead to differences in intestinal microbiota among populations of different regions, and these differences lead to differences in CRC risk among different regions.

This study included a large number of samples worldwide, compared the different intestinal microflora of healthy people and CRC patients in different regions, and identified regional differences in the intestinal microflora of CRC patients. This study provides new ideas for the study of the incidence and etiology of regional differences in CRC.

## Methods

### Data sources and acquisition

CRC-related metagenomic data were collected from GMrepo [31] and NCBI databases (https://www.ncbi.nlm.nih.gov/sra) [32]. The samples with detailed country and age information were screened out, and the samples with missing information such as BMI and age were removed. All WGS sequencing data was uniformly annotated using MetaPhlAn2. The relative abundance table of species level and genus level was obtained for downstream analysis. Samples with less than 2% of the species were removed. Finally, we recruited 601 samples, including 279 CRC samples and 322 healthy controls, as shown in Table 1 and Additional file 1: Figure S1. The quality control steps refer to the article of Wu et al. [31]. The design and workflow of this study are shown in Additional file 2: Figure S2.

**Table 1:** Overview of data information

NCBI project.id	Country	CRC samples	Healthy controls	Total
PRJDB4176	Japan	40	40	80
PRJEB10878	China	72	54	126
PRJEB12449	USA	49	51	100
PRJEB27928	Germany	21	59	80
PRJEB6070	France	51	59	110
PRJEB7774	Austria	46	59	105
		279	322	601

### Descriptive research (composition ratio)

The MicrobiotaProcess package was used to visualize species composition and PCA according to CRC/healthy grouping. The sample composition mainly shows the top 25 species and top 20 genera (in order of species prevalence and average abundance), and the remaining species were classified as others. Second, the logarithmic PCA dimension reduction diagram of the relative abundance of species was calculated. To further display the differences, the differential species of the second part were proposed, and the species composition and PCA diagram were drawn according to the same rules.

### Difference analysis

For the separate difference analysis of each dataset, the species with a prevalence < 0.01 and the maximum relative abundance < 0.001 in all samples were filtered out. LEfSe analysis (http://huttenhower.sph.harvard.edu/galaxy) [33] was performed for the CRC/healthy group for all relative abundance of species composition differences between sample inspection. The threshold value of effect size LDA score (log10) > 2 was used to screen out the differential species, visualize the bar chart of effect size of the differential species (positive and negative only represent the direction, and the magnitude of effect size is the absolute value, and the larger the absolute value is, the greater the difference is), and draw the phylogenetic branching diagram (the maximum level is only labeled to genus).

### Correlation analysis

Spearman correlation coefficients were calculated separately for groups of different disease states, and FDR multiple test correction was performed by BH. Only the correlations with an absolute value of correlation coefficients > 0.3 and P value < 0.05 were retained, and the rest were assigned 0. Plot correlation heatmaps were made using the Corrplot package.

### Construction of the CRC risk prediction model

According to the risk grouping label of the sample phenotype, a binary classifier was established by using the SIAMCAT package [34] three times fivefold nested cross-validation LASSO algorithm. For the training set, species with a maximum relative abundance < 0.001 and mean relative abundance < 0.0001 in all samples were filtered out, and logarithmic standardization was adopted to obtain the internal cross-validation AUC of self-training. In addition, the external validation AUC can be obtained by using the model as external validation with datasets from other regions, and finally, the AUC result graph can be drawn.

### The heatmap describes the difference in bacteria in patients with CRC in different regions

To further highlight the differences in the bacteria of CRC patients in different regions, we selected the species with specific differences in the above regions (LDA Score (LOG10) absolute value > 3 + log2FC > 2 or < -2 species) and the proportion of common bacteria in all samples as pie charts. The MAPS package and the GGploT2 package were used to display the map.

## Results

### The composition of the intestinal bacterial community varies in different regions

**Fig. 1:**
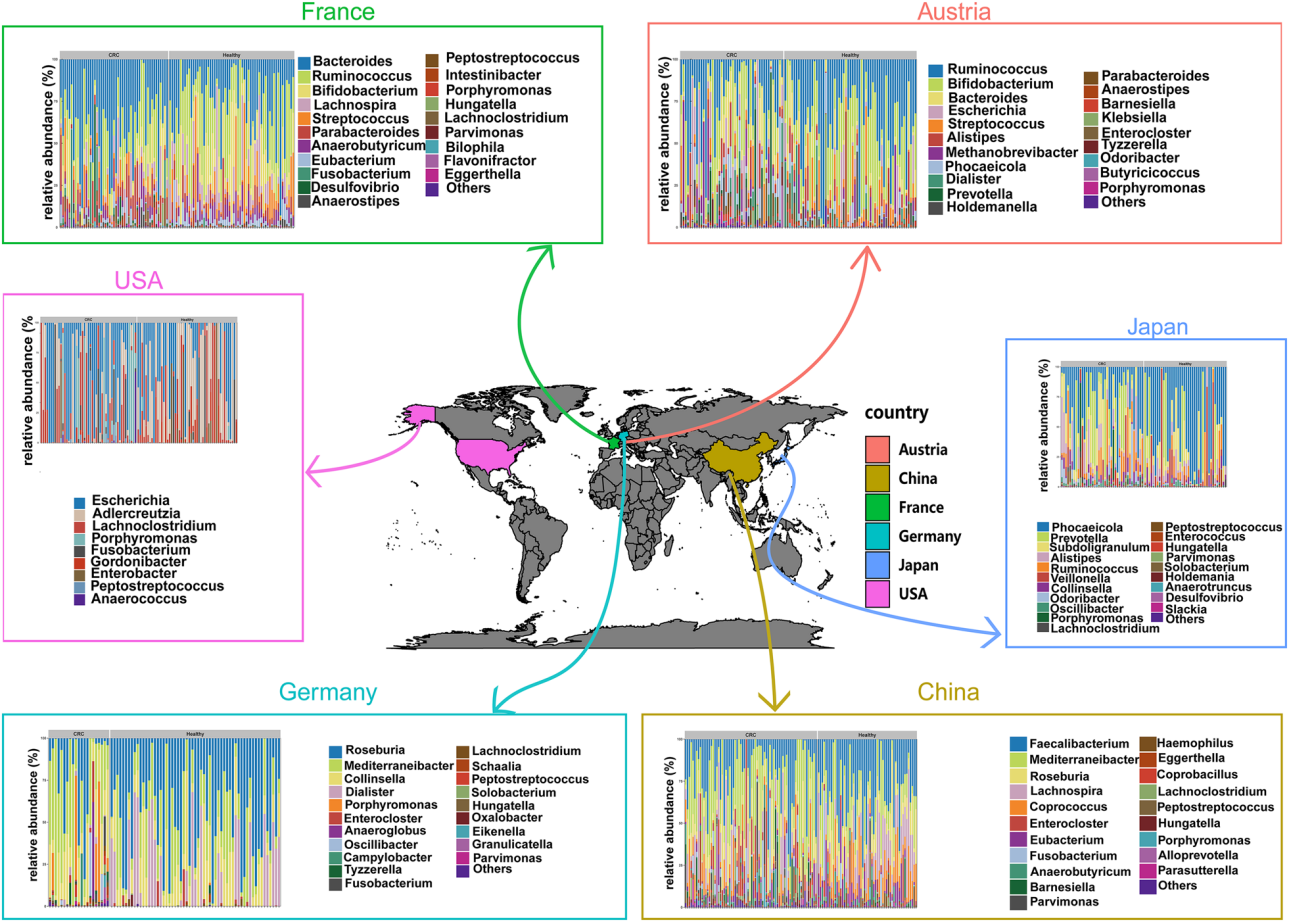
Composition of the intestinal microbiome of populations in different regions. The figure shows the species composition diagram of CRC and healthy people from Japan, China, the USA, Germany, France and Austria

We analyzed the species composition of intestinal bacteria in healthy groups and CRC groups from Japan, China, the USA, Germany, France and Austria at the species level and genus level, respectively. The overall composition of gut bacteria was found to differ between the six countries. For example, at the genus level, *Phocaeicola*, *Prevotella* and *Subdoligranulum* are more prevalent in Japan. *Faecalibacterium*, *Mediterraneibacter* and *Roseburia* are more common in China. *Escherichia*, *Adlercreutzia* and *Lachnoclostridium* are more prevalent in the USA. *Roseburia*, *Mediterraneibacter* and *Collinsella* are more common in Germany. *Bacteroides*, *Ruminococcus* and *Bifidobacterium* are more common in France. There are many *Roseburia*, *Mediterraneibacter* and *Collinsella* in the population of Austria (Fig. 1 and Additional file 3: Figure S3).

At the species level, *Subdoligranulum sp., [Ruminococcus] torques* and *Collinsella aerofaciens* are more prevalent in Japan. *Faecalibacterium prausnitzii*, *Lachnospira eligens* and *Bacteroides caccae.* are more common in China. *Bacteroides ovatus*, *Bacteroides fragili* and *Roseburia hominis* are more prevalent in the USA. *Collinsella aerofaciens*, *Roseburia intestinalis* and *Roseburia inulinivorans* are more common in Germany. *Ruminococcus bromii*, *Lachnospira eligens* and *Bifidobacterium longum* are more common in France. *Ruminococcus sp. 5_1_39BFAA*, *Escherichia coli*, and *Methanobrevibacter smithii* are more prevalent in Austria (Additional file 4: Figure S4).

### There are differences in intestinal bacteria between CRC patients and healthy people in different regions

**Fig. 2:**
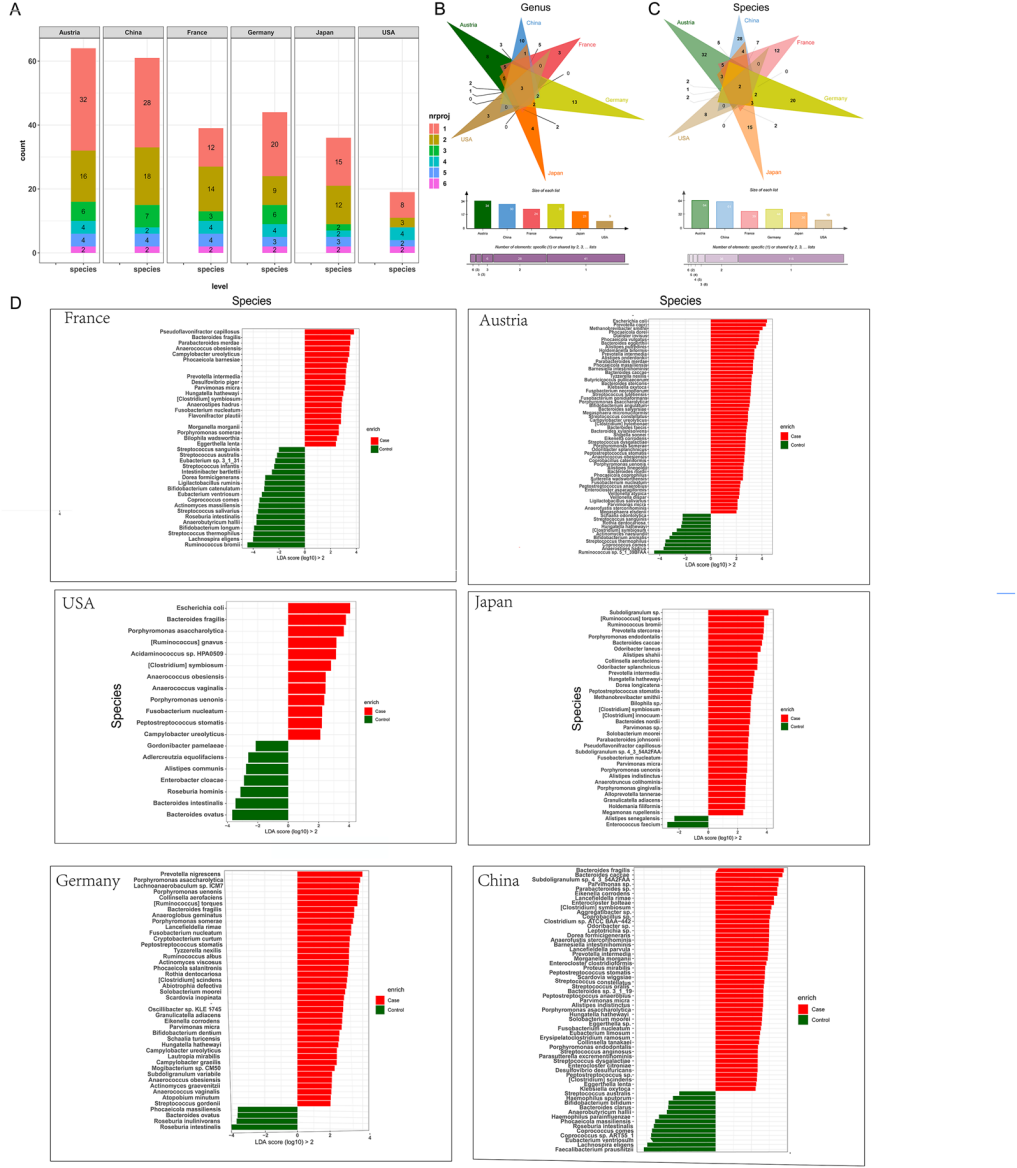
Differences in CRC intestinal bacteria in different regions. Figure **A** CRC differential species frequency statistics of Japan, China, the USA, Germany, France, and Austria at the genus and species levels. Figures **B** and **C** Venn plots of CRC differential intestinal bacteria from six countries at the genus and species levels, respectively. Figure **D** Differential CRC bacteria at the genus and species levels in six countries are described in the map. The LDA discriminant histogram is in the box. The greater the LDA score is, the greater the impact of species abundance on the differential effect

LEfSe analysis was used to test the relative abundance composition of all species among CRC/healthy group samples for the datasets of each country, and it was found that the CRC diversity bacteria were different in different regions (Fig. 2).

In Japan, 36 species CRC differential bacteria at species level, including *Subdoligranulum sp.* and *[Ruminococcus] torque* were found. CRC differential bacteria at genus level, including *Prevotella* and *Subdoligranulum*, of 21 species were identified.

In China, there were 61 types of CRC differential bacteria (*Bacteroides fragilis, Bacteroides caccae*, etc.) at species level and 30 types of CRC differential bacteria (*Mediterraneibacter*, *Eikenella*, etc.) at genus level.

In USA, a total of 19 kinds of CRC different bacteria at species level, including *Escherichia coli* and *Bacteroides fragilis*, and 9 kinds of CRC different bacteria, including *Escherichia* and *Porphyromonas* at genus level were identified.

In Germany, a total of 44 kinds of CRC different bacteria at species level, including *Prevotella nigrescens* and *Porphyromonas asaccharolytica*, and 30 kinds of CRC different bacteria at genus level, including *Mediterraneibacter* and *Porphyromonas* were found.

In France, there were 39 kinds of different bacteria at species level, including *Pseudoflavonifractor capillosus* and *Bacteroides fragili*, and 24 kinds of different bacteria at genus level, including *Bacteroides* and *Fusobacterium*.

In Austria, there were 64 kinds of different bacteria at species level, including *Escherichia coli* and *Prevotella copri*, and 34 kinds of different bacteria at genus level, including *Escherichia* and *Prevotella*.

These results indicate that regional differences affect intestinal microflora.

In addition, we found common bacteria in six countries, including *Peptostreptococcus*, *Porphyromonas* and *Lachnoclostridium* at the genus level (Fig. 2B) and *Peptostreptococcus stomatis* and *Fusobacterium nucleatum* at the species level (Fig. 2C). The USA has the fewest bacteria in common with other regions. The overlap between China and France was highest (Genus: *Eubacterium*, *Lachnospira*, *Anaerobutyricum*. *Morganella*. *Eggerthella*. Species: *Morganella morganii*, *Lachnospira eligens*, *Dorea formicigenerans*, *Eggerthella lenta*, *Streptococcus australis*, *Eubacterium ventriosum*, *Anaerobutyricum hallii*).

**Fig. 3:**
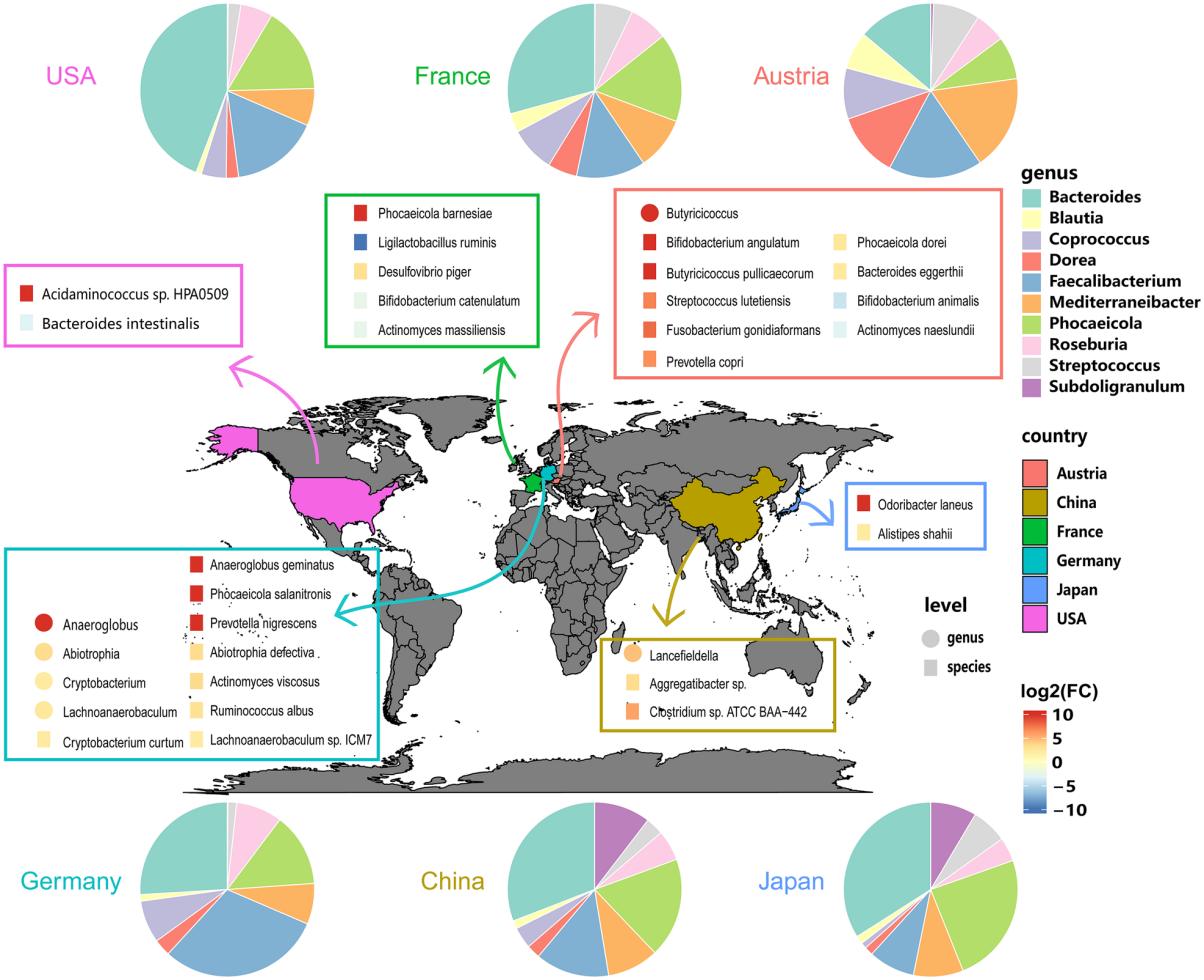
Differences in disease bacteria among different regions. Heatmap describing bacterial differences in CRC patients in different regions. The pie chart shows the percentages of 10 common bacteria at the genus level in six countries

Finally, we screened 10 common bacteria in all the samples*, Bacteroides, Blautia*, *Coprococcus*, *Dorea*, *Faecalibacterium*, *Mediterraneibacter*, *Phocaeicola*, *Roseburia*, *Streptococcus* and *Subdoligranulum*, and calculated the proportion of these 10 bacteria in 6 countries. The results showed that except for in Austria, the proportion of *Bacteroides* was large. *Faecalibacterium* is more prevalent in Germany but less prevalent in other countries. *Subdoligranulum* is only high among people in China and Japan. In addition, we drew a heatmap to visually describe the microflora differences of CRC patients in different regions (Fig. 3). As shown in the figure, CRC-associated bacteria are different in all six countries.

### Interaction of CRC-related intestinal bacteria in different regions

**Fig. 4:**
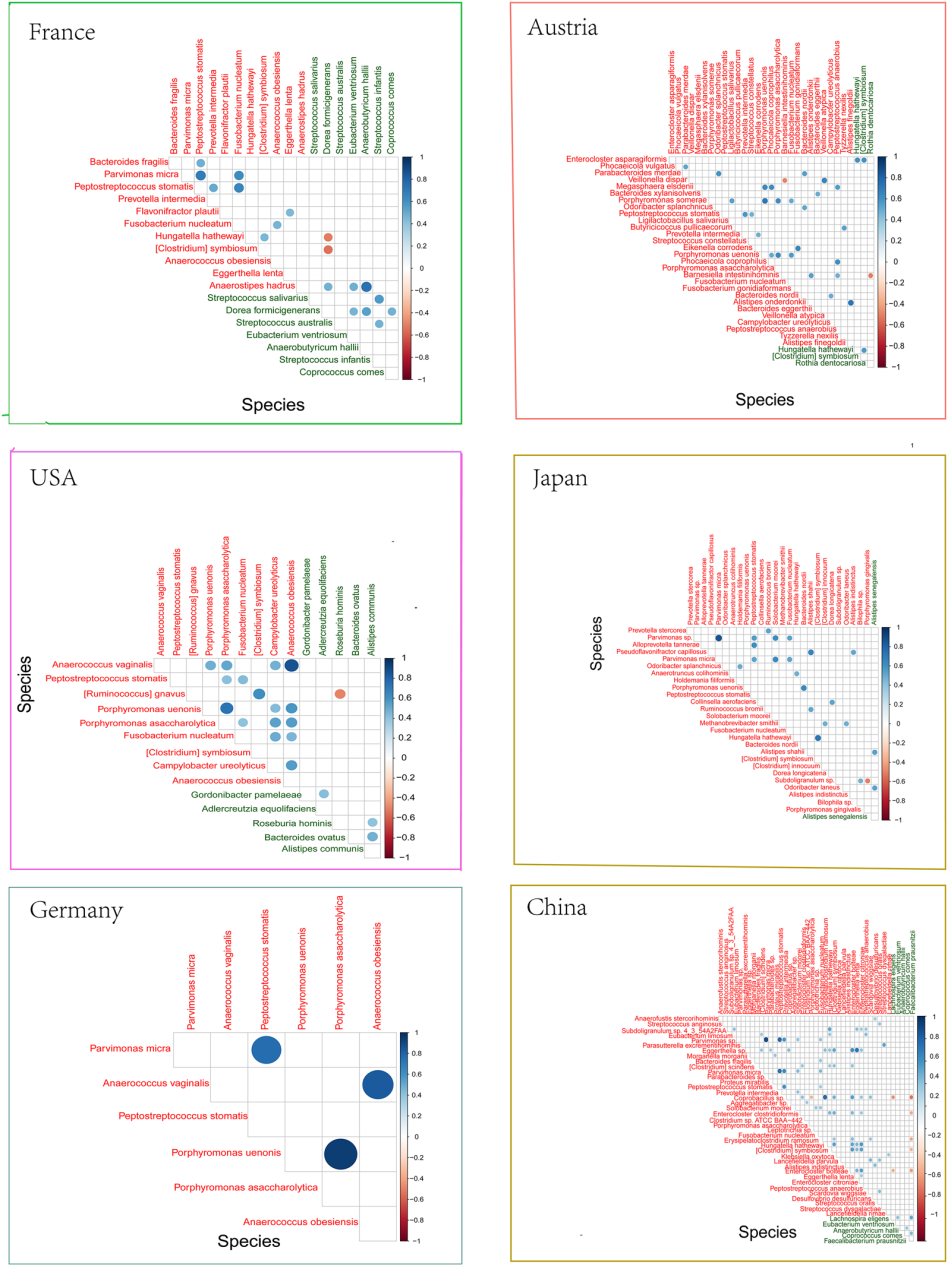
Correlation of different disease-related bacteria. Interactions between differentially abundant bacteria in CRC and healthy groups at the genus and species levels in the map. The dots represent correlations, blue represents positive correlations, and red represents negative correlations. The darker the color and the larger the dots are, the greater the correlation coefficient

In addition, we analyzed the correlation of CRC-differential bacteria in six regions (Figure 4).

At the species level, *Peptostreptococcus stomatis* is an important CRC-related bacterium that is related to other bacteria in different regions. In China, Germany and France, *Peptostreptococcus stomatis* was positively correlated with *Parvimonas sp.* (Spearman=0.78 in China, Spearman=0.77 in Germany, Spearman=0.70 in France). In France, *Peptostreptococcus stomatis* and *Fusobacterium nucleatum* were also positively correlated (Spearman=0.65). Moreover, in Japan and China, *Parvimonas micra* and *Parvimonas sp.* were the most relevant (Spearman=0.86 in Japan, Spearman=0.90 in China).

Similarly, at the genus level, *Peptostreptococcus* was also related to other bacteria. The correlation between *Peptostreptococcus* and *Parvimonas* was high (Spearman=0.84 in Japan, Spearman=0.73 in China, Spearman=0.70 in Germany, Spearman=0.69 in France). In the USA, *Peptostreptococcus* was also associated with *Anaerococcus* (Spearman=0.48).

### Regions do not affect the accuracy of CRC risk prediction models

We constructed CRC risk prediction models based on intestinal bacteria in each region, conducted internal cross-validation and external validation of data from other regions, and ranked the importance of intestinal bacteria in each region model. The final findings are given below.

**Fig. 5:**
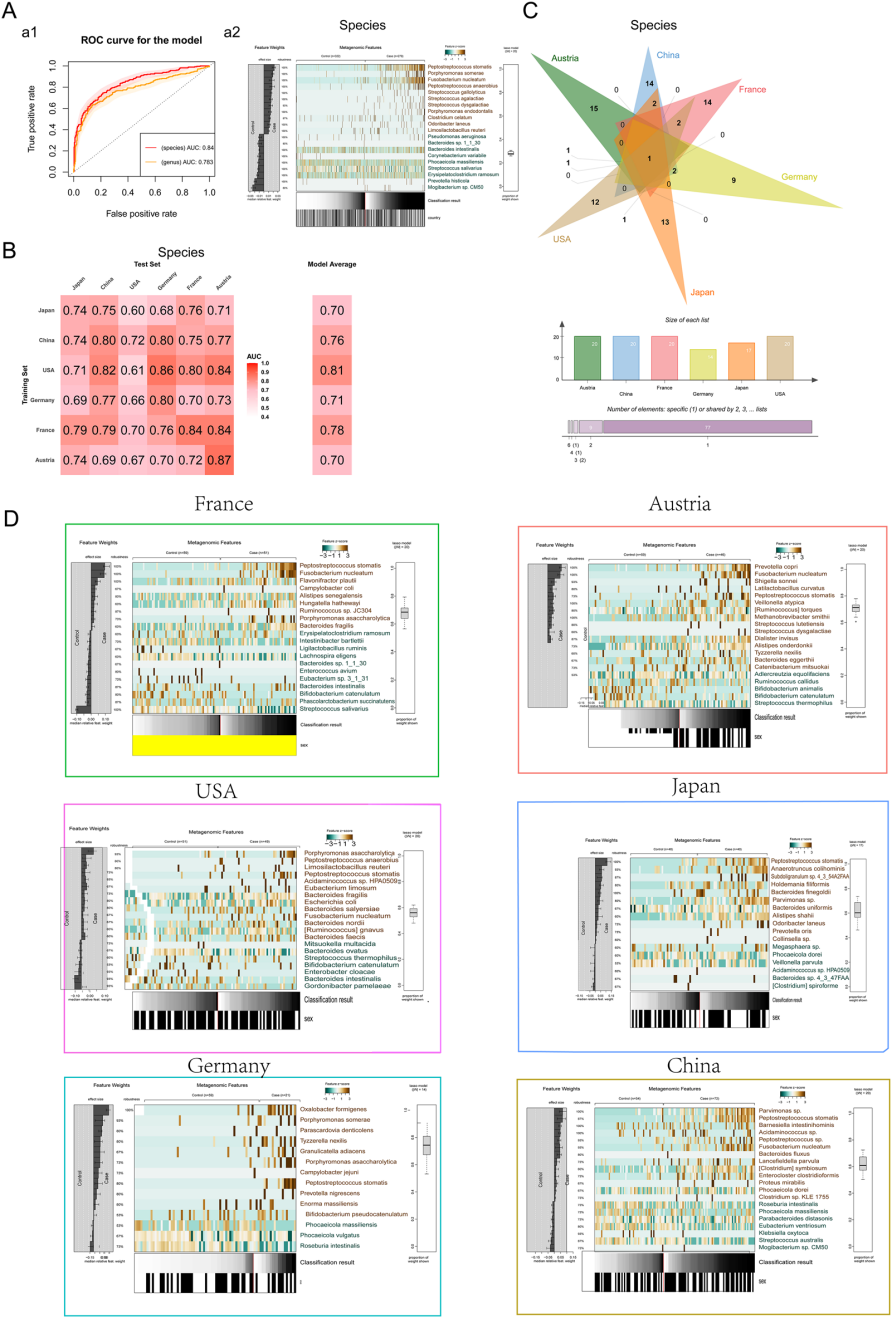
CRC risk prediction model and importance of variables. Figure **A** Overall CRC risk prediction model based on the genus and species levels in all regions. Figure **A**-a1: ROC curve at the genus and species levels. Figure **A**-a2: Top 20 characteristic model interpretation diagrams. The left side represents the relative weights of the corresponding features in the 15 cross-validation submodels in the training model, the middle is the normalized abundance values of each species among the grouped samples, and the right side is the boxplot of the ratio of the 20 features with nonzero coefficients among the 15 submodels. Figure **B** Cross-validation of disease risk prediction models between species datasets in different regions. Figure **C** Venn diagram of the top 20 characteristic bacteria between datasets at the species level. Figure **D** Top 20 characteristic model interpretation diagrams of the species-level CRC risk prediction model in each region in the map

The CRC risk prediction model was constructed based on all regional samples, and the AUCs at the genus and species levels were 0.783 and 0.84, respectively. The intestinal bacteria with the highest importance of CRC risk prediction model variables were *Peptostreptococcus stomatis* at the species level and *Peptostreptococcus* at the genus level (Fig. 5A and Additional file 5: Figure S5B).

Based on the single-region CRC risk prediction model, although its accuracy decreased when applied to other regions, the overall accuracy was within the acceptable range (Fig. 5B and Additional file 5: Figure S5A). Moreover, the accuracy of the CRC risk prediction model based on all regional samples was not lower than that of the single-region model. *Peptostreptococcus* at the genus level and *Peptostreptococcus stomatis* at the species level are still characteristic CRC intestinal bacteria in all regions (Fig. 5C, D).

## Discussion

Based on metagenomic data, this study screened the bacterial characteristics of CRC in different regions and established a CRC risk prediction model. It was found that the composition of the intestinal bacterial community at the species and genus levels was different in the populations of the six countries. CRC-differentiated bacteria were also different in different regions. However, there were also differences in bacteria shared by the six countries, including *Peptostreptococcus stomatis* and *Fusobacterium nucleatum*, at the species level. There are few overlapping intestinal bacteria in the USA with other regions. *Peptostreptococcus stomatis* (species level) and *Peptostreptococcus* (genus level) are important CRC-related bacteria that are related to other bacteria in different regions. There was no significant difference in the accuracy of CRC risk prediction models based on a single region and all regions. The important intestinal bacteria in the CRC risk prediction model are *Peptostreptococcus stomatis* at the species level and *Peptostreptococcus* at the genus level.

*Peptostreptococcus* has been shown to increase significantly in the intestines of CRC patients [35]. Studies have confirmed that the relative species abundance of the oral microbiota in the intestinal tract of CRC patients is significantly increased, including *Peptostreptococcus stomatis*, *Fusobacterium nucleatum* and *Parvimonas micra* [36]. A study on 16S rRNA gene sequencing of intestinal microorganisms in CRC patients showed that there were consistent CRC-related intestinal bacteria in developed and developing countries, and *Parvimonas*, *Peptostreptococcus* and *Fusobacterium* were important in distinguishing CRC from healthy people [37]. Long et al. found that *Peptostreptococcus anaerobius* adsorbed in putative cell wall binding repeat 2 (PCWBR2), targeting the α2/β1-PI3K-Akt-NF-κB signaling axis, which drives CRC [38]. Moreover, Yu et al. confirmed known associations of *Peptostreptococcus stomatis*, *Parvimonas micra*, and *Fusobacterium nucleatum* with CRC [39]. In addition, there was a significant association between *Peptostreptococcus stomatis* and other species, and microbial marker compositions could improve the accuracy of the early diagnosis of CRC [40]. This study analyzed common intestinal bacteria with high CRC correlation in different regions, indicating that *Peptostreptococcus stomatis* is highly carcinogenic to the intestinal tract. However, the carcinogenic mechanism of *Peptostreptococcus stomatis* is not yet known.

In addition, differences were found in CRC-associated bacteria in different regions, and there was little overlap between the USA and the other five countries in CRC differential intestinal bacteria. There are differences between CRC-associated bacteria in China and Japan. The taxonomy and functional composition of the gut microbiome across populations in terms of health or disease are critical to unearthing its role in maintaining human health. Large-scale, global microbiome projects have revealed changes in gut microbiome composition in healthy individuals due to geographic location, host genetics, delivery mode, age, nutrition, diet, and lifestyle [41]. Genetics has been thought to play an important role in determining differences in the microbiome between people. Genes determine the environment the microbiome occupies, and each particular environment allows certain strains of bacteria to grow. Moreover, the diversity and composition of gut microbes vary among different ethnic groups. Deschasaux et al. analyzed the intestinal microbiota of six ethnic groups (439 Dutch, 367 Ghanaians, 280 Moroccans, 197 Turks, 443 African Surinamese, and 358 South Asian Surinamese) living in the Netherlands. Race was found to be an important marker of differences in the composition of human fecal microbiota [42]. However, Rothschild et al. reported that host genetic factors play a minor role in determining the composition of the microbiome, with 98% of the variation in the human gut microbiome determined by environmental factors [43]. A previous study also used metagenomic analysis of the CRC fecal microbiome to obtain microorganisms from CRC cohorts of different races [39]. In addition, different geographical locations within the same country or region can also lead to differences in human gut microbes. For example, Bramble et al. used shotgun metagenomic sequencing to perform a large-scale comparison of gut microbiome profiles in 180 children (from the urbanized capital Kinshasa to extreme rural areas in the southwest of the Democratic Republic of the Congo, including children affected by Konzo disease from prone villages). It was found that the intestinal microbiome structure varied greatly in different regions, but there was no significant difference in intestinal microbiome or functional enrichment between konzo-prone regions [44]. Yan et al. characterized the intestinal microbiota of the population from 14 regions in one province and found that the influence of region on intestinal microbiota was much greater than other factors, and regional differences affected the cross-regional application of disease models [45]. Therefore, the USA is not geographically adjacent to other countries, which may account for the obvious differences in CRC-associated microorganisms between the USA and other regions. Although China and Japan are both Asian countries, there are still differences in CRC-related bacteria. This may be due to the large gap in dietary habits between the two countries, and the main diet structure of Japan includes raw food.

In this study, an overall CRC risk prediction model and a single-region CRC risk prediction model were constructed based on intestinal microorganisms. Although the accuracy of the single-region model fluctuates when applied in other regions, it is within the acceptable range. The accuracies of the multi-region and single-region models are basically the same. In addition, the bacteria common to the six countries identified in this study also play an important role in the classification of the CRC risk prediction model. Based on a CRC metagenomic dataset of 1,368 samples from different geographic cohorts, Liu et al. identified 16 markers in multiple regions (China, Italy, and the United States), including 11 bacteria, 4 fungi, and 1 archaea. The CRC diagnostic model based on microbial characteristics performed well in different geographic cohorts (AUROC = 0.83) [46]. Thus, the prediction of CRC risk using gut microbes does not appear to be affected by the differential bacteria, even though there are differences in CRC-associated bacteria within different regions.

Compared with 16S variable region sequencing of bacteria, metagenomic sequencing can more accurately locate information at the bacterial species level in microbiome studies. Big data provides the basis for this research, indicating that promoting data sharing and making full use of the advantages of big data can promote new discoveries and new meaningful phenomena. Several studies have investigated links between the gut microbiome and CRC through metagenomic data. Thomas et al. performed a meta-analysis of fecal metagenomic datasets from reported cohorts involving five countries and two new cohorts from Italy. The composition and functional characteristics of CRC-associated gut microbiota were identified. CRC prediction model for 16 species was constructed and validated [47]. Wirbel et al. conducted a similar meta-analysis of eight geographically and technically diverse fecal shotgun metagenomic studies of CRC [48]. These two studies confirmed that there was heterogeneity in CRC microbiota characteristics in different populations, but comprehensive analysis of flora markers obtained from multiple cohorts could improve diagnostic accuracy. The difference was that present study highlighted the role of regional differences in CRC gut microbiota, even though the accuracy of our single-region model and multi-region model did not differ significantly.

We collected 601 valid metagenomic data samples of intestinal bacteria from six national datasets (Austria, China, Japan, USA, France and Germany) on three continents (Asia, Europe, and the Americas), deeply explored the differences in intestinal flora in different geographical locations, and further analyzed the relationship between regional differences in intestinal flora and CRC risk. While studies have found that long-term diet, food diversity, and overall nutrition may be important factors in these differences, these ideas have not been proven. This study needs to expand the sample size and include more factors, such as lifestyle, diet and disease, to further analyze the factors related to regional differences in CRC intestinal bacteria.

## Conclusion

In the present study, WGS sequencing data of intestinal bacteria from 601 samples from 6 countries were included and analyzed at the species and genus levels in Japan, China, the USA, Germany, and France. To be confirmed, it was found that the intestinal bacterial community composition and CRC differential bacteria in different regions were different, and regional differences in CRC were identified. In addition, *Peptostreptococcus stomatis* is a CRC-associated bacterium common in all regions. Regional differences in intestinal bacteria had no significant impact on the accuracy of the CRC risk prediction model. In conclusion, region is an important factor leading to differences in CRC-related intestinal bacteria. The study provides new ideas for the study of CRC etiology from the perspective of regional differences.

## Supplementary Information

**Additional file 1: Fig. S1.** Basic information and characteristics of each region.

**Additional file 2: Fig. S2.** The flow chart.

**Additional file 3: Fig. S3.** PCA diagram of CRC and healthy people from different regions. PCA diagram of CRC and healthy people from Japan, China, the USA, Germany, France and Austria. In the PCA dimensionality reduction diagram, the horizontal and vertical coordinates were the first and second principal components (explanatory variances in parentheses), and the top 5 features with the largest contribution to the first and second principal components are shown in the figure.

**Additional file 4: Fig. S4.** Composition of the intestinal bacterial community at the species level in each region

**Additional file 5: Fig. S5.** CRC risk prediction model and importance of variables at the genus level.

## Data Availability

The datasets generated during the current study are not publicly available but obtained from corresponding authors on reasonable request.
