# Supplementary figures and images for: Using whole-genome sequencing (WGS) to plot colorectal cancer-related gut microbiota in a population with varied geography
Source: Gut Pathog. 2022 Dec 28;14:50. doi: 10.1186/s13099-022-00524-x (PMC9795735; doi:10.1186/s13099-022-00524-x)

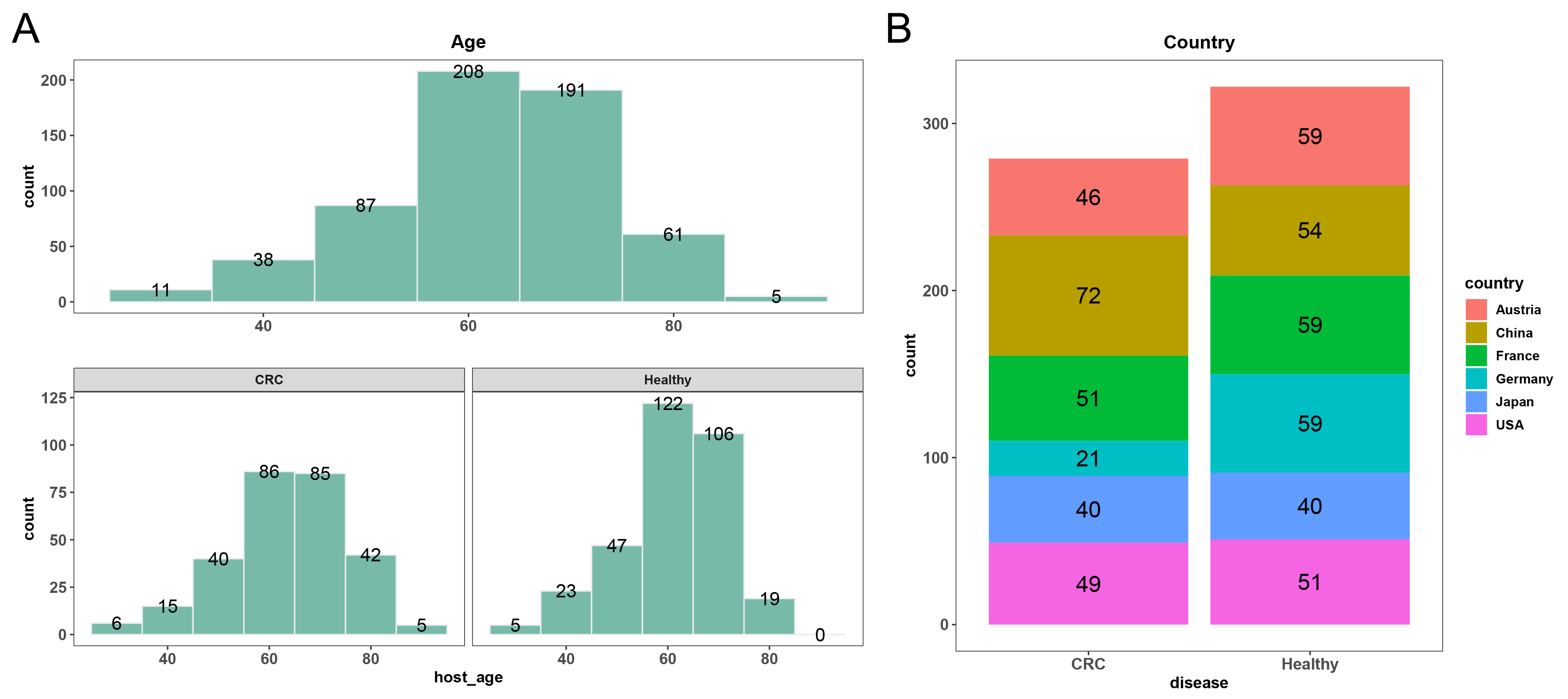

Supplement: Supplementary file 1 — Additional file 1: Fig. S1. Basic information and characteristics of each region. [file 13099_2022_524_MOESM1_ESM.tif]

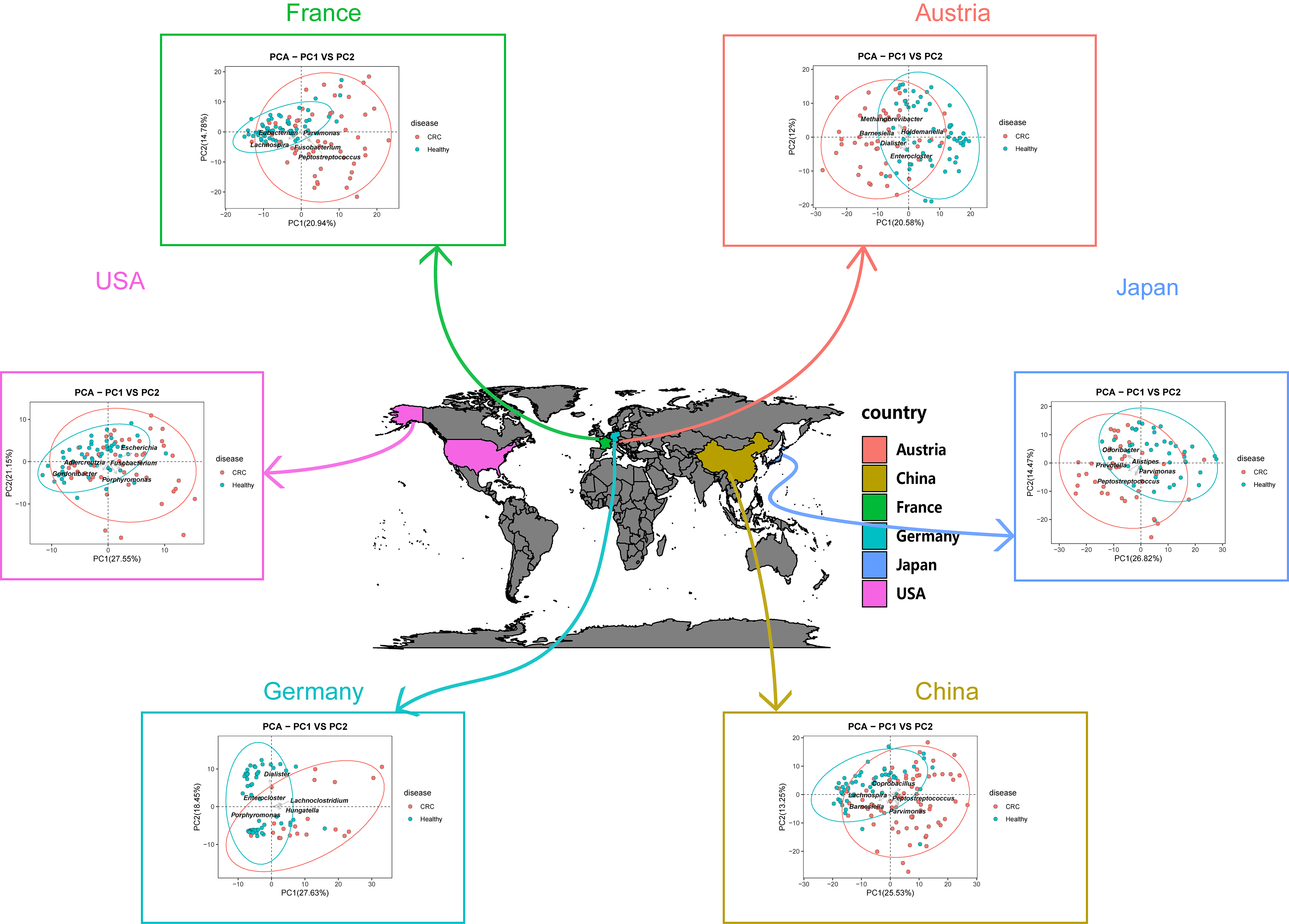

Supplement: Supplementary file 3 — Additional file 3: Fig. S3. PCA diagram of CRC and healthy people from different regions. PCA diagram of CRC and healthy people from Japan, China, the USA, Germany, France and Austria. In the PCA dimensionality reduction diagram, the horizontal and vertical coordinates were the first and second principal components (explanatory variances in parentheses), and the top 5 features with the largest contribution to the first and second principal components are shown in the figure. [file 13099_2022_524_MOESM3_ESM.tif]

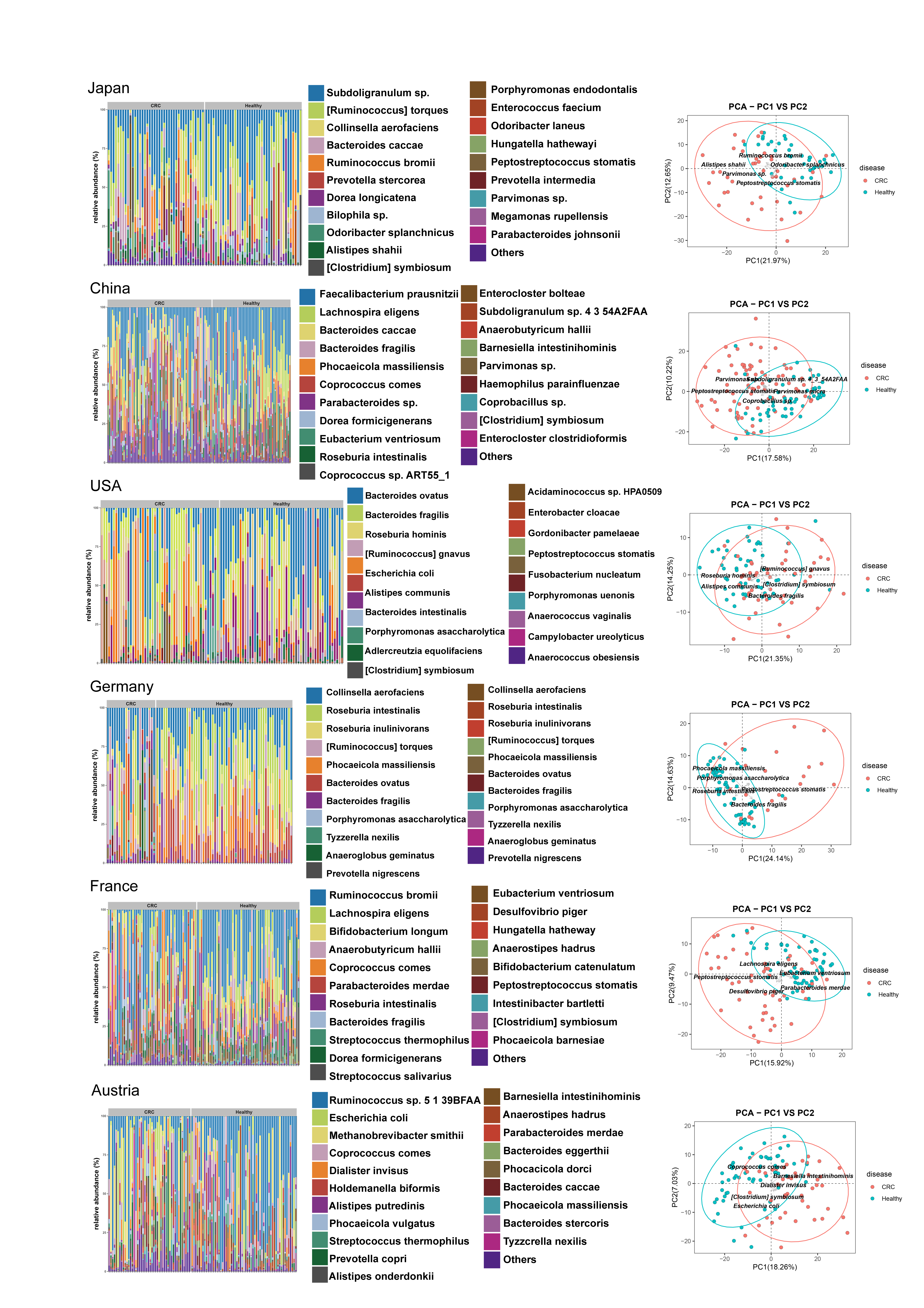

Supplement: Supplementary file 4 — Additional file 4: Fig. S4. Composition of the intestinal bacterial community at the species level in each region [file 13099_2022_524_MOESM4_ESM.tif]

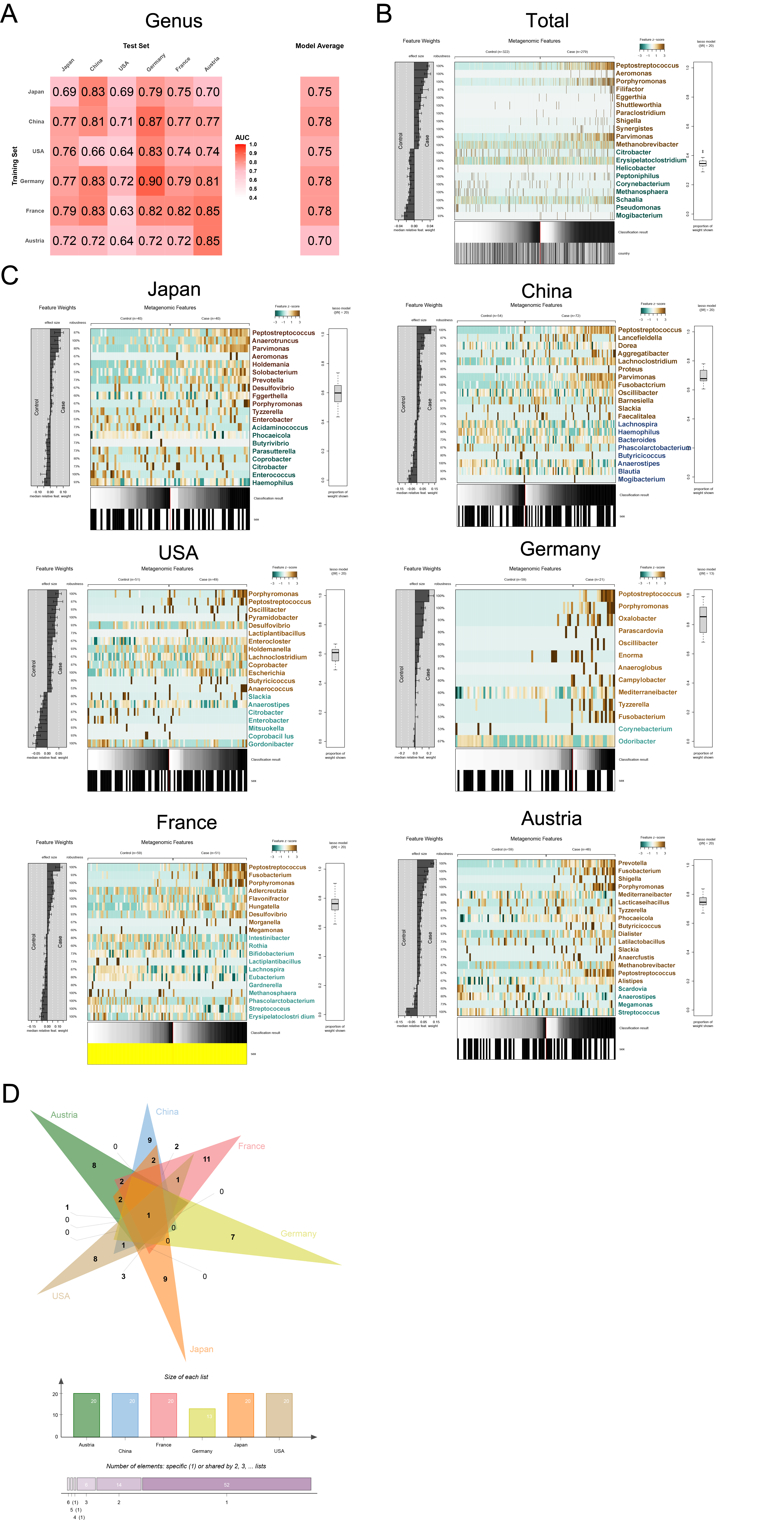

Supplement: Supplementary file 5 — Additional file 5: Fig. S5. CRC risk prediction model and importance of variables at the genus level. [file 13099_2022_524_MOESM5_ESM.tif]
